# Developmental progression continues during embryonic diapause in the roe deer

**DOI:** 10.1038/s42003-024-05944-w

**Published:** 2024-03-05

**Authors:** Anna B. Rüegg, Vera A. van der Weijden, João Agostinho de Sousa, Ferdinand von Meyenn, Hubert Pausch, Susanne E. Ulbrich

**Affiliations:** 1 ETH Zurich, Animal Physiology, Institute of Agricultural Sciences, Zurich, Switzerland; 2 ETH Zurich, Laboratory of Nutrition and Metabolic Epigenetics, Institute of Food, Nutrition and Health, Zurich, Switzerland; 3 ETH Zurich, Animal Genomics, Institute of Agricultural Sciences, Zurich, Switzerland; 4 Present Address: Max-Planck Institute for Molecular Genetics, Berlin, Germany

**Keywords:** Embryonic induction, Differentiation

## Abstract

Embryonic diapause in mammals is a temporary developmental delay occurring at the blastocyst stage. In contrast to other diapausing species displaying a full arrest, the blastocyst of the European roe deer (*Capreolus capreolus*) proliferates continuously and displays considerable morphological changes in the inner cell mass. We hypothesised that developmental progression also continues during this period. Here we evaluate the mRNA abundance of developmental marker genes in embryos during diapause and elongation. Our results show that morphological rearrangements of the epiblast during diapause correlate with gene expression patterns and changes in cell polarity. Immunohistochemical staining further supports these findings. Primitive endoderm formation occurs during diapause in embryos composed of around 3,000 cells. Gastrulation coincides with elongation and thus takes place after embryo reactivation. The slow developmental progression makes the roe deer an interesting model for unravelling the link between proliferation and differentiation and requirements for embryo survival.

## Abstract (web summary)

Changes in epiblast morphology, cell polarity, and correlating gene expression patterns indicate continuous developmental progression during diapause in the roe deer.

## Introduction

Early embryo development of the European roe deer (*Capreolus capreolus*) has dazzled scientists since its discovery in the 19^th^ century^1,2^. While rut and ovulation take place between mid-July to mid-August, embryo implantation only occurs from December onwards^1–5^. Within these 4-5 months, the embryo undergoes embryonic diapause, a period of temporary developmental delay. Diapause emerged to modulate the period between the time of mating and the birth of offspring, thereby adapting to energetic bottlenecks due to environmental and/or metabolic constraints. Both obligate or facultative forms of diapause exist, occurring e.g., seasonally and/or due to lactational inhibition. Among the best-characterised mammalian species displaying diapause are the house mouse (*Mus musculus*) for facultative diapause, the American mink (*Neovison vison*) for obligate diapause, and the tammar wallaby (*Macropus eugenii*), for both facultative and obligate diapause (reviewed in^6^). To date, a reversible arrest of embryo development at the blastocyst stage has been described in over 130 mammalian species across various taxa.

Over the course of diapause, the embryo persists in the uterus as a blastocyst. In the mouse, the reduction of proliferation ultimately leads to a cell cycle arrest at the G1/G0 phase^7^. DNA replication is likewise undetectable in diapausing blastocysts of the rat, the armadillo, and the fur seal^8^. In the mink, more recent evidence^9^ disputes earlier findings^10^ and suggests that DNA replication continues at low rate throughout diapause. In the roe deer, an increase in embryo size during diapause has been documented early on^1,4,5,11,12^. Due to the very low mitotic index^5^, it was initially hypothesised that this size increase is solely caused by cytoplasmic fluid accumulation^13^. However, bromodeoxyuridine (BrdU) incorporation in roe deer blastocysts cultured 4 h in vitro evidenced DNA synthesis – at least from December onwards^14^. We recently demonstrated that the cell number increased slowly and continuously throughout diapause with an estimated duplication rate of 21 days^15^. Proliferation was observed both in the trophectoderm (TE), as well as in the inner cell mass (ICM)/epiblast^15^. With less than 21 days^15^, the rate of proliferation is considerably lower compared to the 3–5 days to be expected in a non-diapausing embryo^16^ and proliferation is not completely halted as seen in other species^7^. The slow proliferation was accompanied by expansion and marked morphological changes of the epiblast^4,15^ including flattening of the initially round ICM, formation of a cavity within the epiblast, and the extension into a disk-like shape^4,15^. The formation of a cell layer facing the blastocoel has been documented already by Keibel in 1902^4^. It was thus postulated that primitive endoderm (PE) formation might occur before or during diapause and that the blastocyst remains bilaminar during diapause in the roe deer^4,6^. These morphological changes, indicative of ongoing differentiation, are in contrast to other diapausing species such as the mouse, the mink, and the tammar wallaby. In these species, the diapausing blastocyst remains unilaminar, thus consisting of ICM and TE only^6,7,17^.

The roe deer is the only ungulate species known to exhibit diapause. A peculiarity of embryo development in ungulates is the extended period between hatching and implantation. In this period, the embryo elongates by rapid trophectoderm expansion^18^, transitioning from a spherical to an ovoid, to a tubular, and finally, to a filamentous shape prior to the epitheliochorial implantation. The elongation of the embryo in roe deer is a morphological hallmark that reactivation has taken place. Compared to the diapause phase, the rate of embryo development and proliferation are thus assumed to drastically increase during elongation, which is supported by the presence of proteins that induce cell proliferation in the uterine fluid^19^. Gastrulation in ungulates is known to occur simultaneously with elongation^20–23^, and thus, in contrast to primates and rodents^24^, prior to implantation. In ungulates, such as cattle and pig, the disappearance of the polar trophoblast is usually observed just prior to or at the beginning of gastrulation^25–30^. Intriguingly, the embryonic disks, observed just prior to elongation in the roe deer, were no longer covered by the polar trophoblast^4,15^.

Roe deer diapause is thus not only characterised by a slow continuation of proliferation, but likely also developmental progression. The present study aimed at enhancing our molecular understanding of developmental processes during embryonic diapause in the roe deer. We used previously obtained RNASeq data from roe deer embryos across diapause and reactivation^31^ and employed an alternative assembly strategy to investigate previously unaccounted for developmentally relevant transcripts.

## Results

### Acceleration of proliferation rate upon elongation

**Fig. 1:**
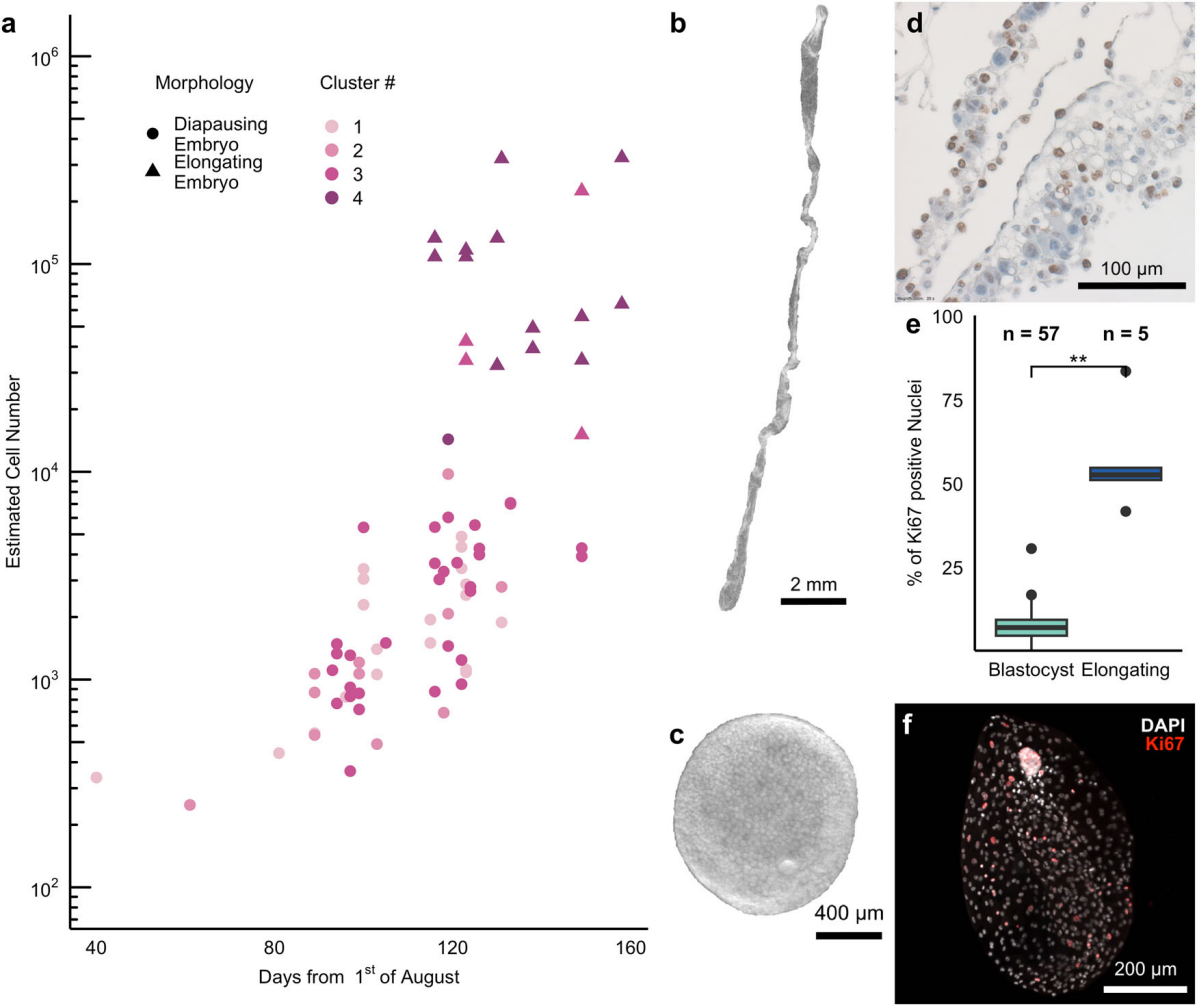
Proliferation rate increases upon embryo elongation. **a** Increase in cell number over time of embryos used for RNASeq. The cell number estimated by the gDNA content is plotted against the the date relative to 1^st^ of August as proxy day of fertilisation. The colour code represents the embryo clusters identified based the expression of marker genes (see also Fig. 2). Source data can be found in Supplementary Table S1. A blastocyst (**c**) and an elongating embryo (**b**) depicted by dark field imaging. **d** Transmitted light image of a section of trophoblast from an elongating embryo stained with KI67 (brown) and haematoxylin (blue). **e** Fraction of KI67 positive nuclei in the trophoblast of blastocysts (*n* = 57) and elongating embryos (*n* = 5) (*p*-value = 0.0027, Kruskal–Wallis test) The boxes display the median value and 25 and 75% quartiles; the whiskers are extended to the most extreme value inside the 1.5-fold interquartile range. Source data can be found in Supplementary Table S2. **f** Representative maximum intensity projection of a blastocyst collected in mid October, stained with DAPI (white) and KI67 (red) imaged using light sheet microscopy.

To confirm the difference in proliferation rates between blastocyst morphology and elongating embryos, we assessed the increase in cell number estimated via the DNA content as well as the fraction of KI67 stained cells. In line with previous studies^4,5,11,15,31^, we found an increase in embryonic cell number over the course of diapause (Fig. 1a and Supplementary Table S1). We confirmed a highly asynchronous embryonic growth across different females as embryos of similar size and cell number were observed during a period of 1-2 months (Fig. 1a). Consistent with our previous report on a different set of embryos^15^, the calculated doubling time for blastocysts was around 21 days. To appreciate the alterations in proliferation rate at different stages of pre-implantation embryo development, we separated our samples into diapausing embryos with blastocyst morphology (Fig. 1c), and reactivated elongating embryos (Fig. 1b). We assessed the fraction of KI67-positive cells on sections of paraffin embedded trophoblast obtained from 5 different elongating embryos (Fig. 1d–f). On average, we found that 57 ± 16% of cells were KI67 positive in elongating trophoblast, which is significantly higher (*p* = 0.002, Welch’s test) than the previously observed 7 ± 5% of KI67 positive cells in the trophoblast of diapausing blastocysts^15^. In an embryonic disc obtained from a single elongating embryo, almost all nuclei were KI67 positive (Supplementary Fig. S2d). Thus, embryos during diapause proliferate at a slow rate and accelerate proliferation upon reactivation.

### Genes within pluripotency and trophectoderm groups positively correlate

To characterise the progression of embryonic development during diapause, we selected 89 frequently used marker genes from a broad spectrum of recent publications (see material and methods). To investigate temporal gene expression changes and developmental progression during diapause, we used RNASeq data of 80 roe deer embryo samples^31^. The abundance of the core pluripotency factors *POU5F1, NANOG* and *SOX2* were strongly correlated (rho ≥ 0.82, *p* < 0.001) with the epiblast markers *OTX2* and *LIN28B* (Supplementary Fig. S1a). These core pluripotency factors also correlated with a group of naïve pluripotency markers including TBX3 and ESRRB (rho = 0.72, *p* < 0.001, Supplementary Fig. S1b). Prominent trophectoderm markers, including *CDX2, ELF5, GATA2, GATA3*, and *DAB2* also significantly correlated with each other (rho ≥ 0.77, *p* < 0.001, Supplementary Fig. S1a, d). The endoderm marker *GATA6* displayed a particularly high correlation with the naïve pluripotency markers *TBX3, KLF5*, and *ESRRB* (rho ≥ 0. *86*, *p* < 0.001, Supplementary Fig. S1a). *TBX3* and ESRRB not only have a prominent role in the maintenance of pluripotency, but have also been associated with extraembryonic endoderm specification^32,33^. We also observed correlations between factors that have not been previously reported to interrelate, such as *GRHL2*, *WNT5A, EMB, SOX17, and STAT3* (rho ≥ 0.77, *p* < 0.001). Interestingly, these mixed group of factors also negatively correlated with markers for extraembryonic endoderm formation (rho = −0.71, *p* < 0.001, Supplementary Fig. S1a, c), as well as with the aforementioned trophectoderm markers (rho = −0.9, *p* < 0.001, Supplementary Fig. S1a, d). For none of the genes analysed, a strong significant correlation between transcript abundance and embryo cell number was observed (rho between −0.45 and 0.60, *p* < 0.05). Within group, the expression of genes characteristic for the earliest two embryonic compartments, the TE and ICM, was highly correlated, indicating that these genes are likely tightly co-regulated and important for embryonic development irrespective of any potential diapause related alteration of embryo morphogenesis.

### Developmental gene expression allows to categorise embryos into distinct groups

**Fig. 2:**
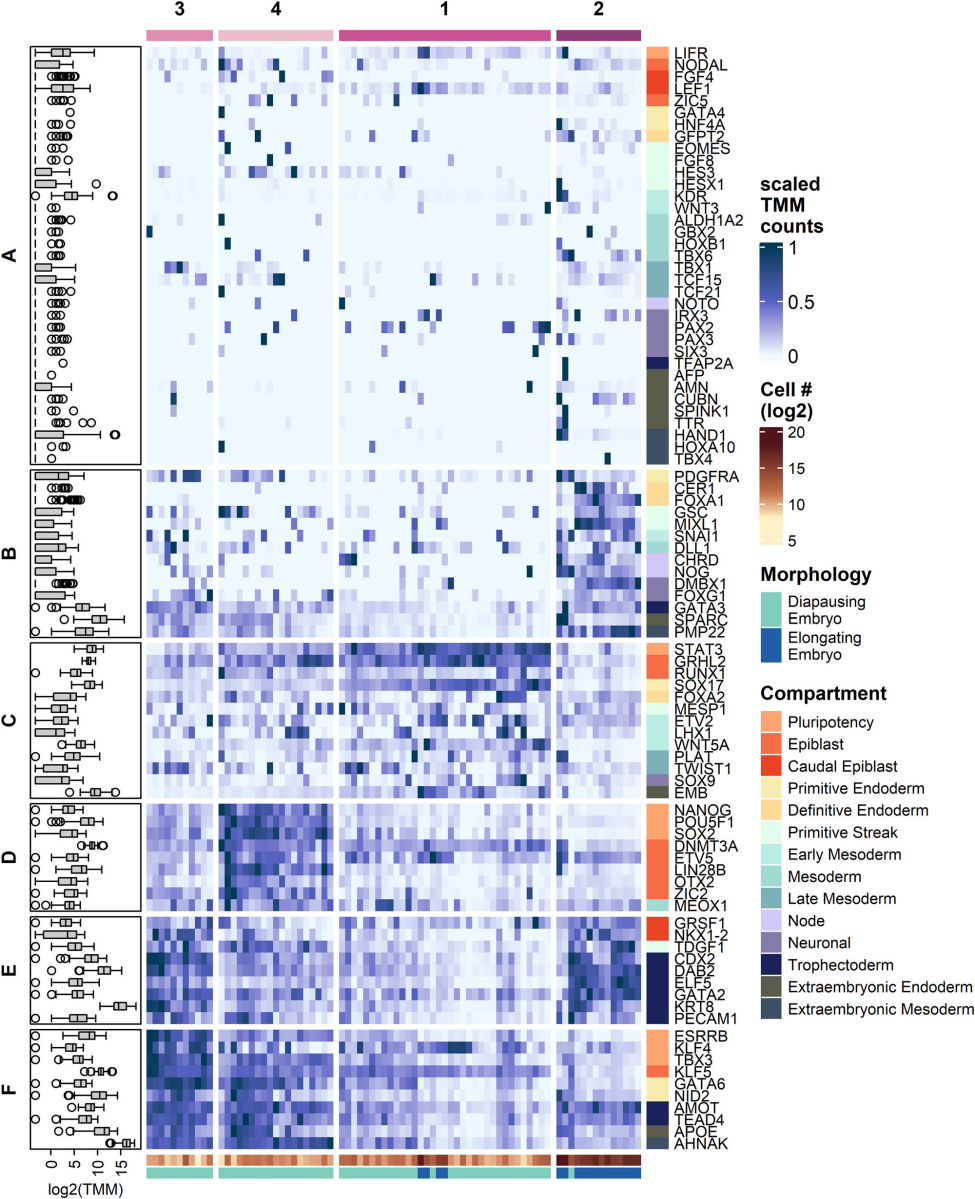
Marker gene expression suggests developmental progression during diapause. Expression patterns of selected genes (rows) in individual embryos (columns). The boxplot on the left indicates the non-standardised gene expression levels. The boxes display the median value and 25 and 75% quartiles; the whiskers are extended to the most extreme value inside the 1.5-fold interquartile range. The heatmap in the centre displays the scaled TMM values. The colour code on top indicates Kmeans cluster # (purples) and the colour codes on the bottom indicate cell number (yellow-orange), and embryo morphology (blue & green). The colour codes on the right indicate the categorisation of genes according to their role in different compartments.

We performed k-means clustering on the transcript abundance data to identify co-expressed genes and substructure in the RNA sequencing data that might temporally resolve developmental progression during developmental pausing. Based on the within-cluster sum of square, we assigned the embryos into four and the selected genes into six clusters with similar expression pattern (Fig. 2, Supplementary Table S1, and Supplementary Data 1). For the embryos, clusters 1 and 2 displayed higher expression levels for genes associated with pluripotency and epiblast development (gene cluster D and F), as well as the majority of trophectoderm markers included in the study (gene cluster F). Embryos in cluster 1 displayed the highest levels of gene expression for core pluripotency factors *POU5F1, NANOG* and *SOX2*, whilst embryos in cluster 2 showed the highest levels of trophectoderm markers, as well as genes potentially linked to extraembryonic endoderm formation.

**Fig. 3:**
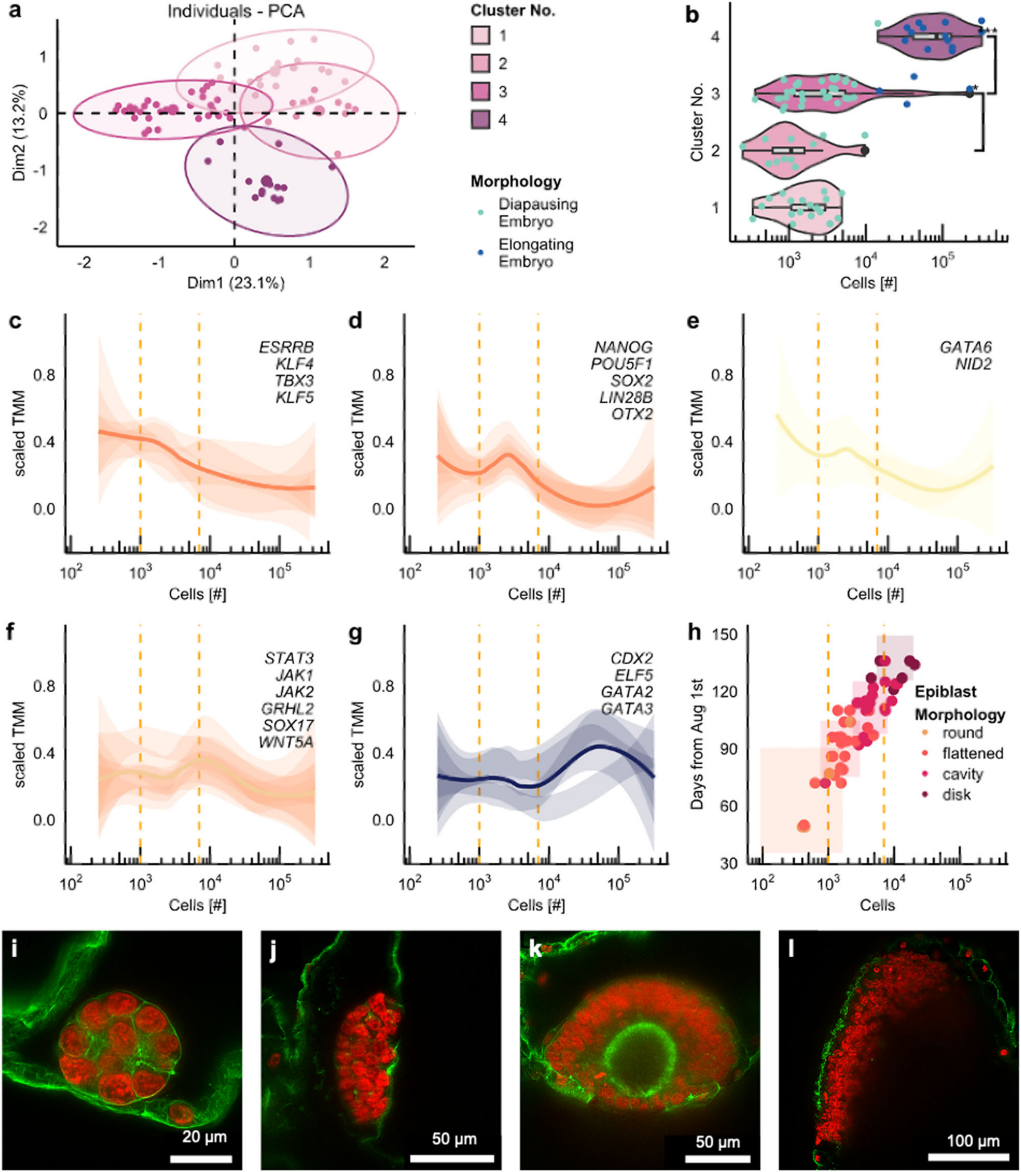
Gene expression patterns translate to morphological changes in the epiblast. **a** PCA plot of the gene expression data of individual embryos. **b** Box and violin plot of cell number for each cluster of embryos formed by Kmeans clustering (two-sided Mann–Whitney–Wilcox test, *** and * indicates *p*-value > 0.001 and >0.05, respectively). The boxes display the median value and 25 and 75% quartiles; the whiskers are extended to the most extreme value inside the 1.5-fold interquartile range. **c**–**g** LOESS smooth of the normalised expression for selected genes as average (line) and of the 95% CI for individual genes (shaded areas) plotted against the cell number. **h** Embryonic age against cell number with colour code according to epiblast morphology. Boxes represent mean +/−SD. Data from previous study^15^. Dashed lines at 1000 and 7000 cells in C-H serve as points of reference. **i**–**l** Representative composite images of individual optical sections of embryos stained with phalloidin (green) and DAPI (red) and classified as either round ((**i**), 05.09., 313 cells), flattened ((**j**), 26.10., 1115 cells), cyst ((**k**), 03.12., 10’627 cells), or disk morphology ((**l**), 06.01., 25’997 cells).

While clusters 1 and 2 consisted solely of diapausing blastocysts, a few elongating embryos were found in cluster 3. Compared to the other clusters, embryos found in cluster 3 expressed elevated levels of genes in gene cluster E: *STAT3, GRHL2*, *SOX17, ETV2*, *WNT5A, and EMB*. All but one embryo in cluster 4 were elongated and displayed higher levels of gene expression for trophectoderm markers compared to the other clusters. Embryos in cluster 4 expressed several genes found in cluster B, some of which are associated with gastrulation (*GSC*, *MIXL1, CER1, DLL)* and node formation (*NOG, CHRD)*. The embryos in this cluster displayed the most distinct expression pattern (Fig. 2, Fig. 3a, and Supplementary Data 1) and had the highest number of cells per embryo (Mann–Whitney–Wilcox, *p* < 0.01, Fig. 3b) compared to the other clusters. Embryos found in clusters 1 and 2 can thus be seen as least, cluster 3 as intermediately and cluster 4 as most advanced in developmental progression, evidencing that embryonic development continues during diapause.

### Gene expression patterns co-occur with morphological changes

While there was no observable linear correlation between any of the investigated genes and cell number, non-linear patterns could still provide valuable insights on developmental progression. We therefore analysed the expression of a subset of marker genes with respect to embryo cell numbers. We found that many of the naïve pluripotency markers included in the study, including *ESRRB, KLF4, TBX3*, and *KLF5*, displayed a rather steady decline as embryo cell numbers increased (Fig. 3c). Core pluripotency marker, as well as epiblast markers, including *POU5F1, NANOG, SOX2, OTX2*, and LIN28B also decreased over development. Yet, their expression profile revealed a distinct peak in embryos comprising around 3000 cells (Fig. 3d). The same pattern was also apparent for *GATA6* and *NID2*, both known to be associated with primitive endoderm formation (Fig. 3e). Interestingly, most of the genes clustering together in cluster E displayed a wave like expression pattern, with the highest expression levels in embryos of either around 1000 or around 7000 cells (Fig. 3f). Genes involved in TE differentiation displayed a rather steady expression until a rise in expression at around 7000 cells (Fig. 3g). Noteworthy, the gene expression alterations coincided with the previously described^15^ morphological transitions in the epiblast (Fig. 3h–l). The observed gene expression patterns might thus be linked to epiblast morphogenesis and indicate ongoing developmental processes.

### Endoderm formation occurs during diapause

**Fig. 4:**
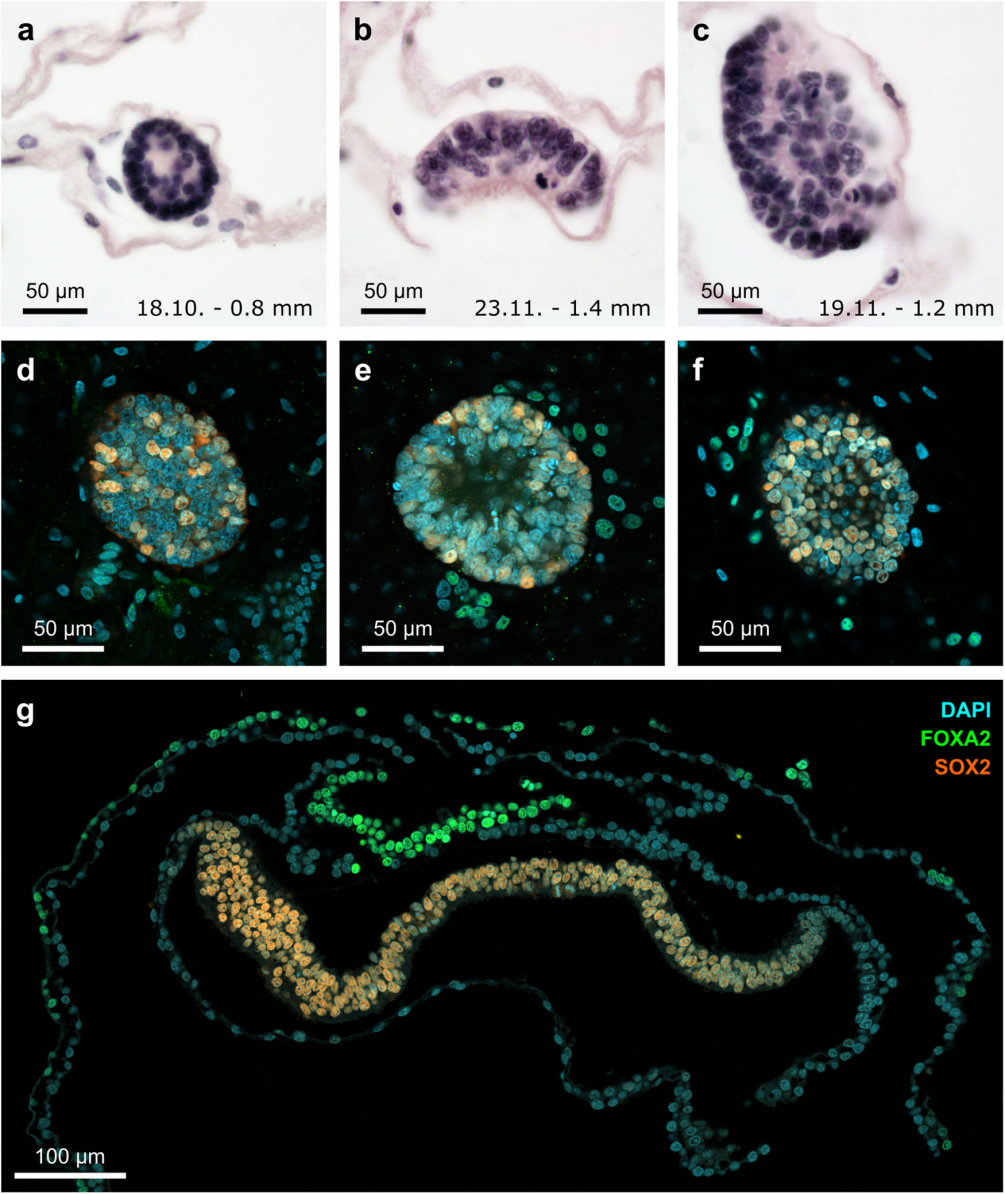
Endoderm formation during diapause. **a**–**c** HE-staining of sectioned blastocysts collected on the indicated dates (DD/MM). Approximate embryo diameter measurements of are indicated. **d**–**g** Expression of the endoderm marker FOXA2 and the epiblast marker SOX2 in sections of epiblasts of late-staged diapausing embryos (**d**)–(**f**) and one elongated embryo (**g**).

Our molecular analyses point to ongoing developmental processes in the diapausing roe deer embryo. Moreover, it has been postulated previously that endoderm formation occurs during diapause^4^. To confirm this finding, we performed H&E-stainings on paraffin embedded blastocysts. In serial sections of blastocyst epiblasts collected between October and November, we were not able to confirm a distinct layer beneath the epiblast (Fig. 4a–c). Nevertheless, we detected *SOX17*-positive cells on blastocyst sections collected in November in four out of nine blastocysts. The expression was mostly limited to nuclei in proximity of the TE but was also found in cells directly adjacent to the epiblast, indicating the presence of a potential hypoblast (Supplementary Fig. S2a–c). Furthermore, we were able to localise the expression of the definitive endoderm marker *FOXA2* in proximity to the epiblast in whole mounts of blastocysts collected in November and early December (8 out of 12), as well as in an elongated embryo collected in December (Fig. 4d–g). Taken together, these results confirm that the primitive endoderm is formed during diapause.

## Discussion

We here provide evidence for developmental continuity during diapause in the roe deer. Next to the fluctuations of transcript abundance found in most genes, we observed trends in gene expression that indicate progressive differentiation. In particular, primitive endoderm formation occurs during diapause and gastrulation occurs upon elongation. Primitive endoderm formation is further supported by the presence and localisation of endoderm markers seen in immunofluorescence. Our alternative assembly and annotation strategy allowed us to include 36 genes (including *POU5F1, OTX2, CDX2 TDGF1)*, which are essential for embryo development but were previously unannotated in the roe deer transcriptome. In line with our earlier study^15^, we calculated a blastocyst cell number doubling time of around 3 weeks (mean of 21 days, 16–30 days using a 95% confidence interval). For this calculation, we assumed August 1^st^ as proxy day of fertilisation, as this information is not available in samples collected from huntings. Despite the relatively high degree of cell number doubling time uncertainty, this time span is extremely long compared to doubling times of well below 24 h in the developing mouse embryo (reviewed in ref. ^34^). We further evidenced a five-fold increase in cell proliferation rate upon reactivation. Since cell cycle durations and phase lengths differ between the cell types and stages of the murine embryo (reviewed in ref. ^34^), it is reasonable to assume that the TE and the epiblast also display different proliferation rates in the elongated embryo.

We hypothesised that the proliferating diapausing embryos continuously develop. Therefore, we analysed the expression of developmentally important genes. Developmental progression is coupled to differentiation and thus exit from pluripotency. We found a transient increase of the core pluripotency factors *NANOG*, *POU5F1*, and *SOX2* in embryos of around 3000 cells followed by a steady decrease. This decrease can be explained by either an increased amount of ongoing differentiation, a reduced proportion of pluripotent cells or a combination of both. With the number of cells in the epiblast being significantly lower than the number of TE cells one might suspect an increasing bias towards TE transcript expression. Yet, up until elongation the proliferation rate for epiblast cells is only marginally lower compared to the trophoblast^15^. This results in a close to constant epiblast/TE ratio, which only substantially shifts upon elongation. A drop in pluripotency related gene expression due to inconsistent epiblast/TE ratios would thus be expected only at elongation. While the relatively low number of embryos available for the immunofluorescence stainings did not allow us to perform a systematic analysis, we indeed observed heterogeneity of the *SOX2* staining intensity within the cells of individual epiblasts. Fluctuations in transcription factor expression, particularly for *NANOG*, are well known to occur in mouse embryonic stem cells. It has been postulated that these fluctuations are important to prime cells for upcoming differentiation steps^35,36^. Thus, while the role of individual pluripotency factors appears to diverge between mice, human and cattle^37,38^, the overall reduction of gene expression for the analysed pluripotency factors points towards ongoing differentiation.

We observed a group of genes (*STAT3, GRHL2, SOX17, ETV2, WNT5A, and EMB – gene cluster E*) displaying a wave-like expression pattern with two distinct peaks at around 1000 and 7000 cells. With the exception of *ETV2*, these genes also significantly correlated with each other. While these genes are not indicative of a particular developmental process or embryonic compartment, many have been linked to cell-polarity related processes. In particular, *GRHL2* has been described to play a role in the epithelialisation of embryonic stem cells into epiblast-like cells^39,40^ loss of *SOX17* disrupts epithelial polarity and adhesion^41^, *EMB* is a cell adhesion molecule potentially involved in epithelial to mesenchymal transition^42^*, and STAT3* and *WNT5A* are known to regulate anterior-posterior axis elongation and as part of the PCP pathway are involved in the regulation of convergent extension and epithelial-mesenchymal transition^40,43^. The WNT/β-catenin pathway has recently been reported to be essential for maintenance of pluripotent cells in the rosette-shaped epiblast during murine diapause^44^. Indeed, the morphology of the actin skeleton during epiblast cavitation in the roe deer strongly resembles the rosette formation observed during rosette formation during pro-amniotic cavity formation in mice^45^. The strong accumulation of f-actin at distinct locations in the epiblast and the non-uniform distribution across the cell membranes points towards ongoing epithelialization and establishment of an apical-basal polarity. Therefore, the regulation of cell polarity could be of importance for the observed morphological changes in the roe deer including symmetry braking from spherical to flattened epiblast, cyst expansion and flattening of the embryonic disc.

The endoderm is the first of the three germ layers and forms at the late blastocyst stage. Due to thorough morphological observations, Keibel et al., suggested more than 100 years ago that endoderm formation occurs prior to or during diapause^4^. The expression patterns of *GATA6 and NID2*, displaying an initial rise of expression peaking at around 3000 cells are in line with and provide molecular evidence for this hypothesis. In addition, our immunohistochemical findings confirming the presence of the primitive endoderm marker *SOX17* and visceral / definitive endoderm marker *FOXA2* in epiblast proximity. The role of *FOXA2* during peri-gastrulation development appears to be well conserved across species^46^. The mRNA expression of *FOXA2* in roe deer embryos was low and heterogeneous and thus did not allow us to draw direct conclusions. Expression of *FOXA2* in the murine extraembryonic endoderm is dependent on the expression of the PE factors *SOX17* and *GATA6* ^47^. Both, *SOX17* and *GATA6* are important for PE specification in cattle^48,49^. The expression pattern of *SOX17* clearly differs from the expression pattern of *GATA6* or *NID2* and its expression rises even further to a peak in embryos of around 7000 cells. Interestingly, *SOX17* has also been reported to be expressed in primitive germ cells in both human and cattle embryos^50,51^. Therefore, the second peak in *SOX17* expression might stem from its function in germ line development. Taken together, we conclude that as previously postulated^4^, hypoblast formation occurs during early diapause stages. In addition, the FOXA2 localisation suggests that differentiation of the extraembryonic endoderm occurs in the later stage blastocysts collected around November.

The elongating embryos display increased expression levels of TE genes, which is most likely linked to the rapid increase in TE cell number. Next to the higher expression levels of TE markers, we also observed an increase of the endoderm markers *FOXA1*^52^ and *CER1*^25,53^, the primitive streak markers *MIXL1*^54^ and *GSC* ^55^, as well as node, notochord, neural crest, and cerebral markers *CHRD*, *NOG* ^56^, *DMBX1*^57^, and *FOXG1*^58^. We thus hypothesise that gastrulation, as well as neurulation events are taking place at elongation.

Considering the importance of transcription factor localisation and signalling factor gradients for embryonic development, our study would have greatly benefited from a single cell-based approach or at least the separation of TE and epiblast. Due to the field sampling setting, we were neither able to perform on site embryo dissections to separate TE and epiblast nor singularise cells for single cell RNASeq. Performing computational cell type deconvolution might have been an alternative solution. However, accurate predictions must rely on adequate single-cell reference not available to date. Due to species-specific differences between rodents, primates, and ungulates^25,37,59–63^, using an existing murine^64–66^ or porcine^67^ dataset would have led to inaccurate predictions. Furthermore, most available single-cell data sets do not cover the extraembryonic tissues. To further disentangle developmental progression during diapause in the roe deer, a single cell RNASeq approach and specific-specific reference atlas are thus invaluable.

Overall, our analyses evidence a continuation of developmental progression during continuous decelerated proliferation over the course of diapause in the roe deer. It thereby contrasts many other diapausing species displaying a complete or almost complete arrest in both^6^. Pluripotency, cell cycle control, and differentiation of stem cells are believed to be tightly co-regulated^68–75^. While the maintenance of pluripotency without proliferation appears puzzling, the two features are uncoupled not only in the diapausing embryo. In fact, cells of diapausing embryos are surprisingly similar to dormant tissue stem cells, and therapy resistant dormant cancer cells^76–78^. Pre-implantation development in roe deer thus represents a model for very slow developmental progression. This offers a unique opportunity to investigate determinants of differentiation and developmental timing. Furthermore, it provides a prolonged window for studying embryo-maternal communication during developmental stages, which are often difficult to obtain in other species. The roe deer holds promises for improving our understanding of the regulation of developmental pace and the coordination between maintenance of pluripotency, differentiation, and proliferation.

## Methods

We re-analysed a previously obtained RNASeq dataset^31^. A schematic overview of the methods used is depicted in Supplementary Fig. S3a. All custom code was deposited on https://github.com/Animal-Physiology-ETH/roe_deer_dev_diapause^79^.

### Sample collection for nucleic acid extraction and RNA sequencing

Sample collection, nucleic acid extraction and RNA sequencing have been reported earlier^31^. In brief, roe deer reproductive tracts were obtained from regular huntings over the course of three consecutive hunting seasons between September and January of 2015/16, 2016/17, and 2017/18 (see Fig. 1). No ethical approval was required, since all samples were collected at regular huntings from carcass remnants irrespective of the research question. Uteri were kept on ice after retrieval, dissected and flushed with phosphate buffered saline (PBS) to obtain the embryos. These were visualised under a Zeiss SteREO Discovery.V8 microscope and imaged using an Olympus SC-50 camera. After imaging, embryos were washed in PBS, snap-frozen in liquid nitrogen, and stored at −80 °C. All embryos were frozen within 8 h after the animal was shot. A total of 81 embryos from 56 does were subjected to nucleic acid extraction using the Qiagen AllPrep DNA/RNA micro kit and library preparation using the Clontech pico-input mammalian total RNA kit. RNA sequencing was performed on a NovaSeq (single-read 101 base pairs) at the Functional Genomics Centre Zürich (FGCZ). The average sequencing depth obtained was 20 million reads per sample. The genomic DNA content was determined using the Promega QuantiFluor® ONE dsDNA System to estimate the number of embryonic cells. Data of one embryo was excluded from further analyses due to very low DNA content. The doubling time was calculated as ln(2)/k, where k is the slope of the linear regression line for the log-transformed number of cells over the course of time (in days from first blastocyst collected).

### Retrieval of reference transcripts for embryo development genes

De-novo transcript assembly was performed for 678 developmentally relevant genes. These genes included all genes of the gene ontology term GO0048598 - embryonic morphogenesis as well as genes selected based on a broad array of publications. These publications consisted of experimental studies^64,65,80–82^ and review articles on mouse^46,83–86^, cattle^25–27,87–89^ and pig embryo development^55,90^. Orthologs from selected species (mouse, human, bovine, goat horse, pig, sheep) were collected based on the gene name and the cDNA sequences. These were retrieved from Ensembl^91^ using biomaRt^92^ version 2.48.2. The multi species reference transcripts were later used to assemble the roe deer reference transcripts.

### Assembly, annotation and quantification of embryo development genes in roe deer

RNASeq reads from all 80 embryos were used for the roe deer reference transcript assembly. Low quality regions and adapters were removed using Trim Galore! version 0.4.3.1^93^ with the following parameter settings: phred33, quality 30, stringency 1 -e 0.1, length 50, output_dir ./, no_report_file, input_1.fastq, dont_gzip. The cleaned-up reads were then mapped to the set of multi species reference transcripts using HISAT2 version 2.1.0^94^ (x ’genome’, U ’input_f.fastq’, rna-strandness R). The mapped reads were then extracted and assembled using Trinity version 2.4.0.2^95^ with default settings. Of the selected 678 developmentally relevant genes, 603 (89%) were successfully assembled.

Those genes were further filtered to only keep the ones with the highest identity score to annotated homologues in mouse, human, and bovine. The selected genes were then merged into a previously assembled roe deer reference transcript that included developmental and non-developmental genes^31^. Gene sequences with high similarity between assemblies were flagged using BLAST 2.8.1+^96^ with the following parameter settings: -outfmt 6 -max_target_seqs 1 -evalue 0.05 -perc_identity 90. Then, only the BLAST flagged gene sequences from the developmental genes reference transcript assembly were kept in the merged roe deer reference transcript assembly, together with the non-flagged gene sequences. The read quantification was performed using the Trinity version 2.14.0 script *align_and_estimate_abundance.pl* with RSEM version 1.3.3^97^ as the abundance estimation method, bowtie2 version 2.4.4^98^ as the alignment method, and the merged roe deer reference transcript assembly as the transcript file.

### Statistics and reproducibility

A subset of 89 genes (Supplementary Fig. S3b), selected based on their biological relevance during embryogenesis, was analysed in more detail. The genes were categorised according to function and presence in embryonic compartments that develop during these stages: pluripotency, epiblast, caudal epiblast, primitive endoderm, definitive endoderm, primitive streak, early mesoderm, mesoderm, late mesoderm, node, neuronal, trophectoderm, extraembryonic endoderm, and extraembryonic mesoderm. Statistics and downstream analyses were performed in R version 4.2.0. Normalisation of RSEM expected counts was performed using edgeR 3.38.1^99^ without any filtering. The correlation matrix was generated from the normalised transcript abundance estimates using corrplot version 0.88. Spearman correlations were calculated and the cut-off for displaying a value was set to a *p*-value of 0.05. The correlation matrix was sorted using hierarchical clustering. Optimal numbers of clusters for k-means clustering were determined using the weighted sum of squares method (wss) implemented in factoextra version 1.0.7. For heatmaps, TMM values were min-max scaled [(x − min_gene_)/(max_gene_ − min_gene_)] and visualisation was done using ComplexHeatmap^100^. The principal component analysis (PCA) individual plots were created using factoextra. All other plots were generated using ggplot2 based packages^101^. Illustrations and arrangement of subfigures was done in Inkscape 1. The normalized and scaled expression data depicted in Figs. 2 and 3, can be found in Supplementary Data 1. All custom code can be found in the following repository https://github.com/Animal-Physiology-ETH/roe_deer_dev_diapause^79^.

### Embryo collection for microscopy

Embryo collection for microscopy and imaging was performed as previously described^15^. In brief, embryos used for staining were collected during huntings from September 2018 to December 2021. Uteri were flushed and imaged as described in the section on the extraction of nucleic acids above. After imaging, embryos were washed in PBS + 0.1% poly-vinyl-alcohol (PBSP). For fixation, embryos were incubated for 30 min in 3.7% paraformaldehyde (PFA) in PBS. Prior to storage at 4 °C in PBS-P + 1% Penicillin-Streptomycin, embryos were washed for 10 min in PBS-P. For long-term storage embryos were transferred to 70% ethanol or 3.7% PFA.

### Paraffin embedding

For paraffin embedding and subsequent sectioning, blastocysts stored in 70% ethanol were rehydrated by incubating in 50% and 30% ethanol in PBSP for 10 min each. After rehydration, ethanol, as well as PFA stored blastocysts were washed three times in PBSP for 3 minutes and once for at least 1 h. Blastocysts were then embedded in drops of 2.5% low-melting-point agarose at 42 °C. Agarose blocks were cut to cuboids and dehydrated through an ethanol series. Samples were kept in individual glass vials and a sieve was used to prevent sample loss during liquid exchange. Before transfer to Tissue-Tek^®^ Paraform^®^, samples were incubated twice for 30 min in xylene. Samples were transferred to a new vial containing Paraform^®^ and kept at 60 °C. Paraform^®^ was exchanged two more times – after 1 h and >12 h before embedding using a Leica EG1150 embedding station. 10 µm sections were cut using a Leica HistoCore AUTOCUT rotary microtome, transferred to a 37 °C water bath and mounted on either untreated glass slides for haematoxylin-eosin (HE)-staining or SuperFrost™ slides for immunofluorescence (IF) and immunohistochemistry (IHC). Elongated embryos used for sectioning were treated identically, apart from the low-melting-point agarose embedding steps.

### Immunofluorescence and Immunohistochemistry

Slides were deparaffinised, rehydrated and washed under running tap water followed by 5 min incubation in citrate buffer (1.8 mM citric acid monohydrate, 8.2 mM trisodium citrate dihydrate, pH 6.0) at room temperature (RT). Then, slides were boiled three times in citrate buffer for 5 min using a Severin MW7869 microwave at around 600 W. The samples were allowed to cool for 20 min at RT and washed for 5 min under running tap water. For IHC, slides were incubated for 30 min in 0.3% hydrogen peroxide in methanol. For IF, this step was omitted. Slides were washed for 5 min PBS + 0.1% Tween20 (PBST) before being covered with a Shandon™ cover plate and mounted into a Sequenza™ staining rack. After washing two more times for 5 min in PBST, slides were incubated for 60 min in blocking buffer (2.5% bovine serum albumin [Sigma-Aldrich] in PBST). All primary anti-bodies (Supplementary Table S4) were diluted in blocking buffer and incubated over night at 4 °C. Slides were washed three times with PBST before incubation with secondary antibodies (Supplementary Table S4) diluted in blocking buffer. For IF, 4′,6-Diamidin-2-phenylindol (DAPI) was added at a final concentration of 1 to 2 μg/ml to the secondary antibody mix. After washing three times for 5 min in PBST, slides were de-mounted from the Shandon™ cover plates. For IF, coverslips were directly mounted using VECTASHIELD® H-1000, and edges were sealed with nail polish. For IHC, slides were incubated for 2-5 min with diaminobenzidine using the ROTI®DAB kit. After washing for 10 min under running tap water, slides were dipped in to acidic hemalum solution acc. to Mayer and blued for 5 min under running tap water. Slides were dehydrated in an ascending ethanol series (see above) and coverslips were mounted using Eukitt™.

For whole mount immunofluorescent staining of blastocysts followed by confocal imaging, embryos were washed 2 times for 10 minutes and once for 30 min in PBST and then subjected to the IF staining procedure. Blastocysts were mounted using VECTASHIELD® within a ring of previously dried Eukitt™.

### Phalloidin staining

Seventeen blastocysts with 401-25,997 cells that had previously undergone IF for KI67^15^ were randomly selected for an additional phalloidin staining. Embryos were manually dissected from the agarose and any remaining agarose was dissolved using 60°C PBSP. Embryos were washed twice in PBSP + 0.1% Tween20 (PBSTP) and incubated for 45-60 minutes at RT in Phalloidin-iFluor 488 Reagent, diluted 1:1000 in PBSTP. Embryos were then washed twice in PBSTP, nuclei were re-stained by incubating in 1 ug/ml DAPI in PBSP for 10 min, washed twice in PBSP and re-mounted in mounted in 1.2% LMP agarose at 42 °C.

### Microscopy, imaging, and image processing

Light sheet microscopy was performed at the centre for microscopy of the University of Zurich (ZMB) using a Zeiss Lightsheet - Zeiss Z.1 microscope equipped with 405 nm (20 mW) and 561 nm (50 mW) solid state diode lasers. The filter sets dapi-draq5 (lp 490, bp 420–470, lp 660) and gfp-mcherry (lp 560, bp 505–530, lp 585) were used. The 20x W Plan APO objectives was used and images were captured using two 3.8 M pixel sCMOs-Cameras. Image acquisition and microscope control was done using the ZEN Black Light sheet software. Confocal microscopy was performed at ScopeM using a Zeiss LSM 780 microscope, equipped with 2 conventional PMTs, a 40x water immersion objective (LD C-Apochromat 40x/1, M27), the following laser lines: diode 405 nm, argon: 458, 477, 488, 514 nm and solid state: 561 nm and was operated using ZEN Black 2012. Brightfield images were obtained using a Zeiss AxioPlan microscope equipped with an Olympus DP73 camera, operated via cellSens Standard. Regions of interest were selected, and stacks were cropped using ZEN or ImageJ.

### Reagents

Lists of reagents and antibodies used, including suppliers, antibody dilutions, and article numbers are given in Supplementary Tables S2 and S3, respectively.

### Reporting summary

Further information on research design is available in the Nature Portfolio Reporting Summary linked to this article.

### Supplementary information

Supplementary Material

Description of Additional Supplementary Files

Supplementary Data 1

Reporting Summary

## Data Availability

The raw transcriptome data analysed in this article has been previously published^31^ and is accessible in NCBI’s Gene Expression Omnibus (GEO) through GEO Series Accession No. GSE158806. The source data is found in Suppl Data 1.

## Code availability

All custom code can be found in the following repository https://github.com/Animal-Physiology-ETH/roe_deer_dev_diapause^79^.
