# Supplementary material for: Developmental progression continues during embryonic diapause in the roe deer
Source: Commun Biol. 2024 Mar 5;7:270. doi: 10.1038/s42003-024-05944-w (PMC10914810; doi:10.1038/s42003-024-05944-w)
Supplement: Supplementary file 1 — Supplementary Material [file 42003_2024_5944_MOESM1_ESM.pdf]

Developmental progression continues during embryonic diapause in the roe deer

Running Title: Developmental progression in roe deer

Anna B. Rüegg[1], Vera A. van der Weijden[1,#], João Agostinho de Sousa[2], Ferdinand von Meyenn[2], Hubert Pausch[3], Susanne E. Ulbrich[1,✉]

Supplementary material

Figure S1

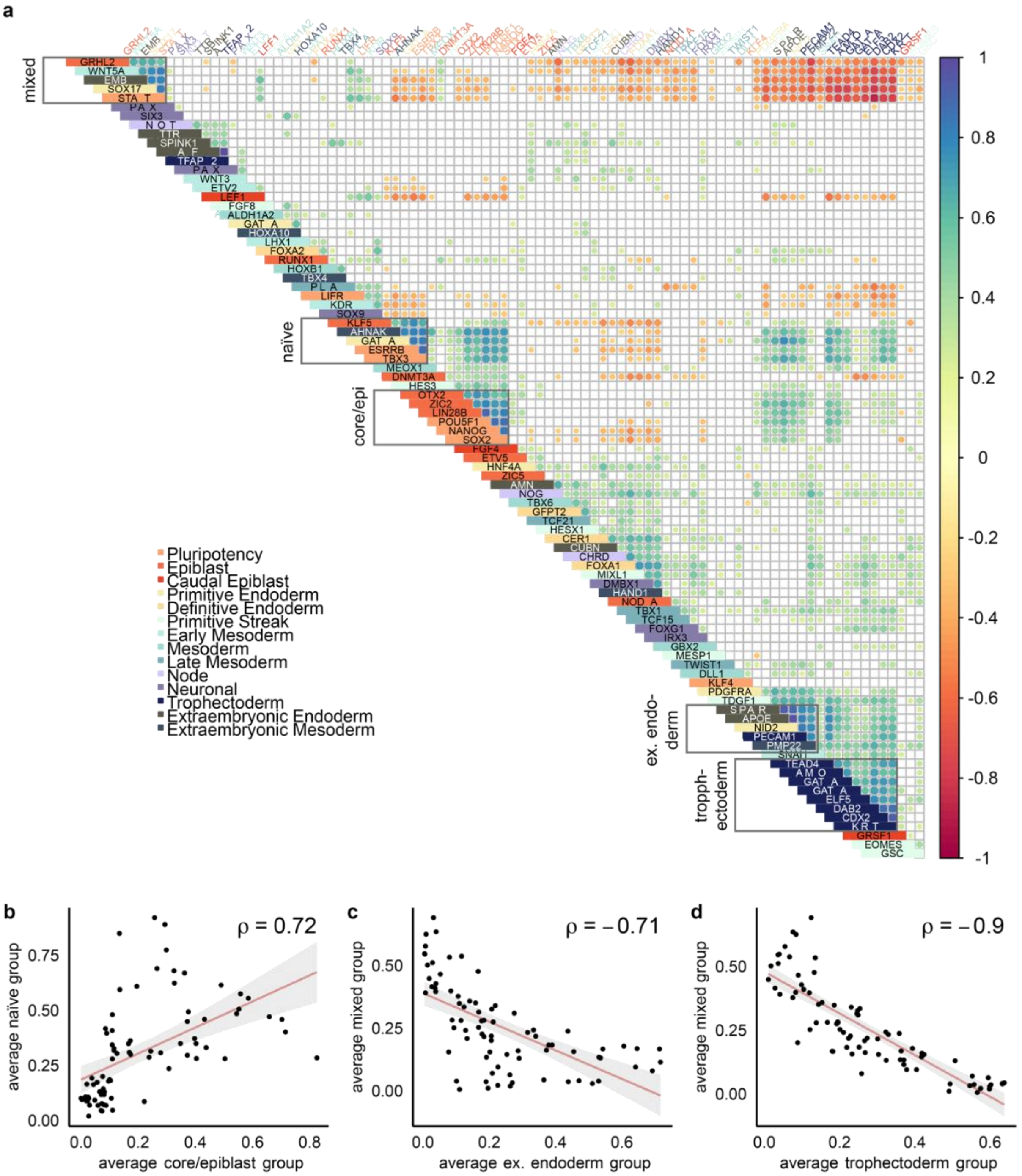

Fig. S1: Correlation of transcript abundances. a) Correlation matrix provides an overview of correlations between all genes under analysis. The colour code used in gene names reflects stages of embryonic development in which the gene is particularly important. Gene groups displaying particularly high correlations (rho > 0.7) are highlighted using boxes. b-c) Strong correlations occurred between different highly correlating groups.

Figure S2

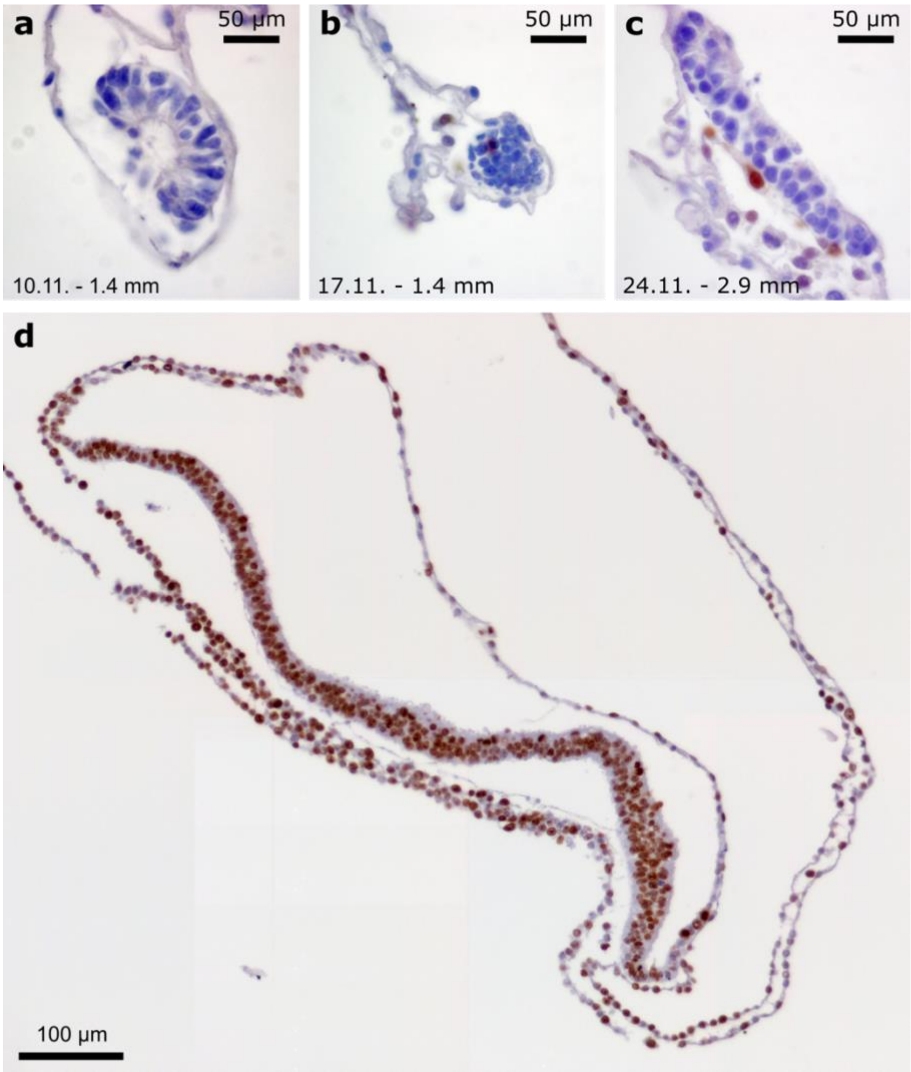

Fig. S2: Immunohistochemistry displaying *SOX17* and *KI67* positive cells (brown). a-c) Staining for *SOX17* on blastocyst collected on the indicated dates (DD/MM). Approximate diameter measurements are also given. a) embryo without positive staining, b and c) embryos with *SOX17* positive nuclei in epiblast proximity. d) Staining of *KI67* on section through the embryo proper of an elongated embryo.

Figure S3

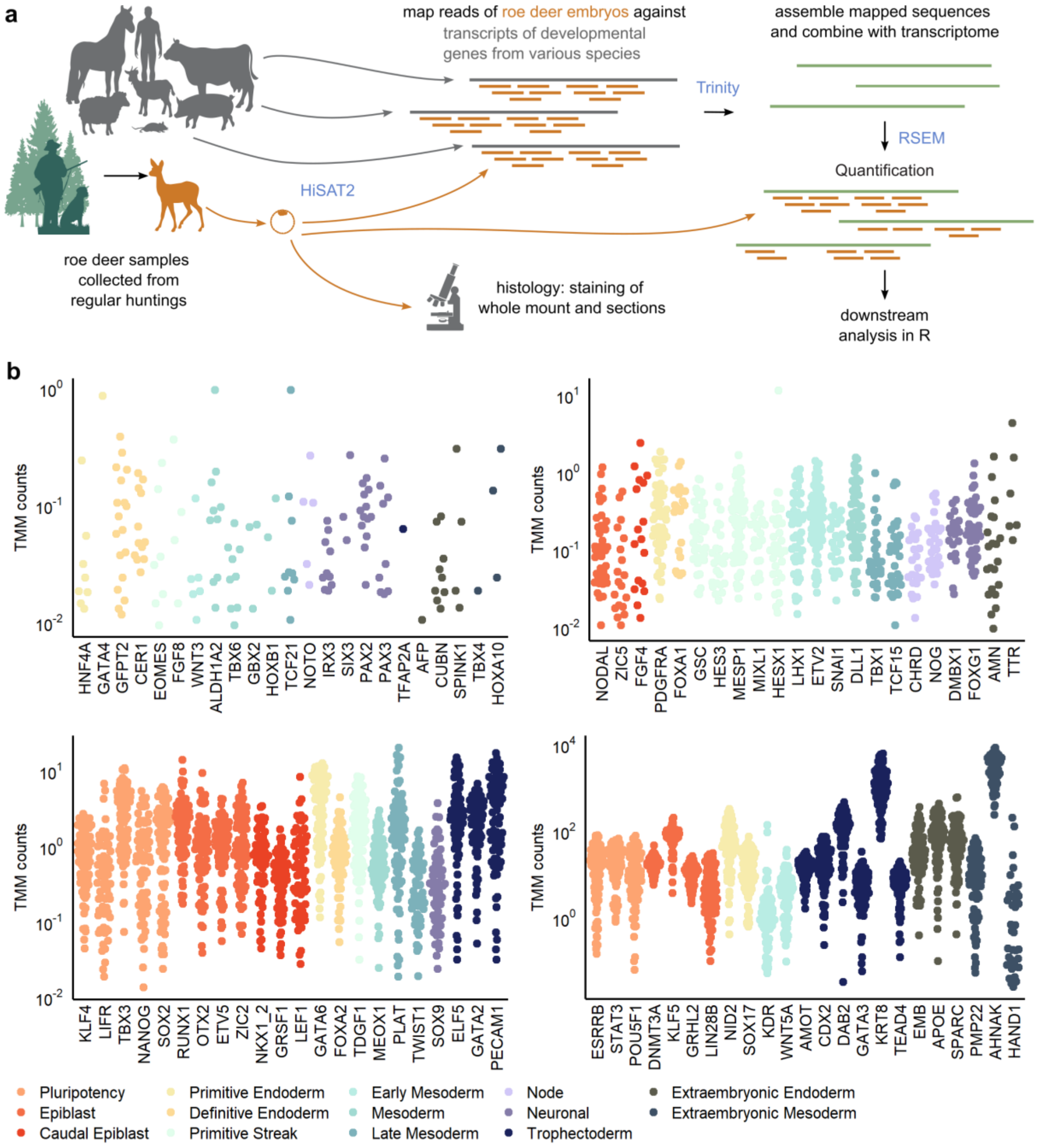

Fig. S3: Methodological approach and overview on gene expression levels. a) Graphical overview of method used to generate a partial transcriptome for the roe deer. b) Overview of absolute expression levels for individual genes analysed.

Supplementary Tables

Supplementary Table S1: Metadata of embryos used for RNA-Seq. Date refers to the date of sample collection. Cell numbers are estimated based on DNA-content. Diameter is given in micrometre. Cluster Number refers to the clusters generated by K means (see Figure 2).

Embryo ID	Roe deer ID	Collection date	Group	Embryo diameter [µm]	Embryo cell number [#]	Cluster number
E1041_e1	104	2016-10-21	Diapause	531	442	1
E1081_e1	108	2016-10-29	Diapause	915	1067	2
E1082_e1	108	2016-10-29	Diapause	646	867	2
E1091_e1	109	2016-10-29	Diapause	453	552	1
E1101_e1	110	2016-10-29	Diapause	567	541	2
E1141_e1	114	2016-11-03	Diapause	881	1333	3
E1142_e1	114	2016-11-03	Diapause	758	1483	3
E1181_e1	118	2016-11-08	Diapause	1449	1208	2
E1182_e1	118	2016-11-08	Diapause	1141	1067	2
E1191_e1	119	2016-11-08	Diapause	1128	718	3
E1192_e1	119	2016-11-08	Diapause	865	858	3
E1231_e1	123	2016-11-03	Diapause	979	768	3
E1281_e1	128	2016-11-05	Diapause	833	826	1
E1311_e1	131	2016-11-12	Diapause	1568	489	2
E1321_e1	132	2016-11-12	Diapause	1712	1058	1
E1322_e1	132	2016-11-12	Diapause	1412	1400	1
E1401_e1	140	2016-11-24	Diapause	737	1500	1
E1402_e1	140	2016-11-24	Diapause	482	1942	1
E1421_e1	142	2016-11-25	Elongated		108333	4
E1422_e1	142	2016-11-25	Elongated		133333	4
E1591_e1	159	2016-11-26	Diapause	1717	3033	3
E1681_e1	168	2016-11-28	Diapause	2472	2075	2
E1721_e1	172	2016-11-28	Diapause	4283	14333	4

E1722_e1	172	2016-11-28	Diapause	1293	9750	2
E1821_e1	182	2016-12-02	Diapause	1409	1083	1
E1822_e1	182	2016-12-02	Diapause	11280	1117	1
E1831_e1	183	2016-12-01	Diapause	1835	4875	1
E1832_e1	183	2016-12-01	Diapause	1791	4350	1
E1861_e1	186	2016-12-01	Diapause	1221	3433	1
E1871_e1	187	2016-12-01	Diapause	1086	1242	3
E1872_e1	187	2016-12-01	Diapause	840	950	3
E1901_e1	190	2016-12-02	Elongated	7903	42500	3
E1902_e1	190	2016-12-02	Elongated	4934	34381	3
E1911_e1	191	2016-11-09	Diapause	1859	2292	1
E1912_e1	191	2016-11-09	Diapause	1986	3050	1
E1921_e1	192	2016-11-09	Diapause	1879	5400	3
E1922_e1	192	2016-11-09	Diapause	1859	3408	1
E1931_e1	193	2016-12-09	Elongated	18399	32500	4
E1932_e1	193	2016-12-09	Elongated	17878	133333	4
E1941_e1	194	2016-12-10	Elongated	7399	321429	4
E2011_e1	201	2016-12-02	Diapause	1563	2875	1
E2012_e1	201	2016-12-02	Diapause	1211	2550	1
E2031_e1	203	2016-12-02	Elongated	24958	116667	4
E2032_e1	203	2016-12-02	Elongated	18033	108333	4
E2091_e1	209	2016-12-10	Diapause	800	1883	1
E2092_e1	209	2016-12-10	Diapause		2792	2
E212_e1	21	2015-11-02	Diapause		1108	3
E2131_e1	213	2016-12-28	Elongated	6000	225000	3
E2181_e1	218	2016-12-28	Elongated	6000	34500	4
E2182_e1	218	2016-12-28	Elongated	6600	55833	4
E2191_e1	219	2016-12-28	Elongated	2700	15083	3
E2221_e1	222	2016-12-28	Diapause	1500	4292	3

E2222_e1	222	2016-12-28	Diapause	1800	3917	3
E2251_e1	225	2017-01-06	Elongated	40000	325000	4
E231_e1	23	2015-11-06	Diapause	1200	362	3
E281_e1	28	2015-11-06	Diapause	1300	1308	3
E291_e1	29	2015-11-06	Diapause	1100	917	3
E301_e1	30	2015-11-06	Diapause	700	830	3
E321_e1	32	2015-11-14	Diapause		1500	3
E381_e1	38	2015-11-25	Diapause	1800	3625	3
E382_e1	38	2015-11-25	Diapause	2600	5417	3
E401_e1	40	2015-11-25	Diapause	860	875	3
E472_e1	47	2015-11-27	Diapause	1600	692	2
E491_e1	49	2015-11-28	Diapause		1450	3
E501_e1	50	2015-11-28	Diapause		6033	3
E511_e1	51	2015-11-27	Diapause		3308	3
E591_e1	59	2015-11-30	Diapause		3650	3
E631_e1	63	2015-12-03	Diapause	1100	2792	3
E632_e1	63	2015-12-03	Diapause	1200	2667	3
E691_e1	69	2015-12-04	Diapause		5542	3
E721_e1	72	2015-12-05	Diapause	1240	4275	3
E722_e1	72	2015-12-05	Diapause	1300	3992	3
E741_e1	74	2015-12-12	Diapause	3000	7008	3
E781_e1	78	2015-12-17	Elongated		39167	4
E782_e1	78	2015-12-17	Elongated		49167	4
E801_e1	80	2016-01-06	Elongated	4000	64167	4
E811_e1	81	2015-12-12	Diapause	2500	7083	3
E881_e1	88	2016-09-10	Diapause	430	338	1
E991_e1	99	2016-10-01	Diapause	654	249	2

Supplementary Table S2: Data underlying Figure 1e.

Sample ID	Number of Ki67 + cells	Embryo cell number [#]	Group	Collection date
Doe417_E1	19	313	Blastocyst	2018-09-05
Doe421_E2	14	401	Blastocyst	2018-09-19
Doe421_E1	31	441	Blastocyst	2018-09-19
Doe447_E1	66	427	Blastocyst	2018-09-20
Doe433_E2	32	628	Blastocyst	2018-10-12
Doe433_E1	63	897	Blastocyst	2018-10-12
Doe433_E3	90	1560	Blastocyst	2018-10-12
Doe436_E1	120	1242	Blastocyst	2018-10-16
Doe438_E1	148	1034	Blastocyst	2018-10-17
Doe439_E1	242	1660	Blastocyst	2018-10-19
Doe440_E1	148	1538	Blastocyst	2018-10-20
Doe444_E2	35	1115	Blastocyst	2018-10-26
Doe445_E1	54	1172	Blastocyst	2018-10-26
Doe443_E1	142	1782	Blastocyst	2018-10-26
Doe453_E1	164	2896	Blastocyst	2018-11-01
Doe456_E1	8	1572	Blastocyst	2018-11-02
Doe458_E1	44	1928	Blastocyst	2018-11-03
Doe459_E1	4	2605	Blastocyst	2018-11-03
Doe459_E2	250	3849	Blastocyst	2018-11-03
Doe470_E2	3	1151	Blastocyst	2018-11-05
Doe478_E1	107	1387	Blastocyst	2018-11-05
Doe478_E2	5	1576	Blastocyst	2018-11-05
Doe476_E1	262	3432	Blastocyst	2018-11-05
Doe462_E2	445	4866	Blastocyst	2018-11-06
Doe466_E1	325	3964	Blastocyst	2018-11-10
Doe483_E2	5	1734	Blastocyst	2018-11-13
Doe483_E1	31	2170	Blastocyst	2018-11-13
Doe494_E1	2	1392	Blastocyst	2018-11-19
Doe496_E1	213	2108	Blastocyst	2018-11-19
Doe491_E2	9	2135	Blastocyst	2018-11-19
Doe508_E2	177	3745	Blastocyst	2018-11-19
Doe493_E1	294	4154	Blastocyst	2018-11-19
Doe490_E2	405	7375	Blastocyst	2018-11-19
Doe501_E1	290	3870	Blastocyst	2018-11-20
Doe498_E2	182	7227	Blastocyst	2018-11-20
Doe518_E1	338	2741	Blastocyst	2018-11-23
Doe507_E2	122	2860	Blastocyst	2018-11-23

Doe506_E1	285	4312	Blastocyst	2018-11-23
Doe520_E1	428	3710	Blastocyst	2018-11-24
Doe520_E2	356	4075	Blastocyst	2018-11-24
Doe521_E1	667	9197	Blastocyst	2018-11-24
Doe523_E1	215	4667	Blastocyst	2018-11-27
Doe524_E2	939	9821	Blastocyst	2018-11-30
Doe526_E2	295	4798	Blastocyst	2018-12-01
Doe527_E1	1650	10627	Blastocyst	2018-12-03
Doe527_E2	849	11296	Blastocyst	2018-12-03
Doe529_E1	684	7304	Blastocyst	2018-12-04
Doe532_E1	305	4488	Blastocyst	2018-12-06
Doe531_E2	945	13251	Blastocyst	2018-12-06
Doe540_E1	6293	20546	Blastocyst	2018-12-13
Doe555_E1	134	6101	Blastocyst	2018-12-15
Doe542_E1	510	6817	Blastocyst	2018-12-15
Doe555_E2	409	7280	Blastocyst	2018-12-15
Doe558_E1	2901	17259	Blastocyst	2018-12-15
Doe578_E1	485	6493	Blastocyst	2018-12-29
Doe575_E2	1400	14800	Blastocyst	2018-12-29
Doe592_E1	3731	25997	Blastocyst	2019-01-06
Doe675_E2	479	937	Elongating	2019-11-28
Doe699_E2	581	1103	Elongating	2019-12-14
Doe675_E1	488	891	Elongating	2019-11-28
Doe695_E1	551	659	Elongating	2019-12-11
Doe699_E1	521	1248	Elongating	2019-12-14

Supplementary Table S3: List of reagents used, including supplier and article numbers.

Chemical/reagent	Supplier	Article No.
Potassium chloride for analysis	Merck KGaA	104933
Sodium chloride for analysis	Merck KGaA	106404
Potassium dihydrogen phosphate	Sigma-Aldrich	1.04873
di-Sodiumhyrogenphosphate-Dihydrate	Merck KGaA	1.06580.1000
Qiagen AllPrep DNA/RNA micro kit	Qiagen	80284
QuantiFluor® ONE dsDNA System	Promega	E4871
Poly(vinyl alcohol)	Sigma-Aldrich	P8136
Paraformaldehyde	Sigma-Aldrich	P6148
Penicillin-Streptomycin (10,000 U/mL)	ThermoFisher	15140122
Tween 20	Sigma-Aldrich	P1379
Bovine Serum Albumin	Sigma-Aldrich	A9647
Phalloidin-iFluor 488	Abcam	ab176753
Citric acid monohydrat	Merck KGaA	100244
Diamidin-2-phenylindol (DAPI)	Sigma-Aldrich	D9542

low melting point agarose	Sigma-Aldrich	A9414
Ethanol (≥99,8 %, denatured)	Carl Roth	K928.4
Xylol (Isomere)	Carl Roth	9713.1
Tissue-Tek® Paraform®	Sakura Finetek	7052
Citric acid monohydrate	Merck KGaA	100244
Sodium citrate tribasic dihydrate	Carl Roth	4088.3
Hydrogen peroxide, 35 wt.% solution in water	ACROS organics	A0361650
Methanol	Sigma-Aldrich	32213
VECTASHIELD® Antifade Mounting Medium	VECTOR Laboratories	H-1000
ROTI®DAB Kit	Carl Roth	9202.1
Hemalum solution acid acc. to Mayer	Carl Roth	T865.1
Eukitt quick-hardening mounting medium	Sigma-Aldrich	3989
Eosin Y	Carl Roth	7089.1

Supplementary Table S4: List of antibodies with suppliers, article numbers, as well as dilutions used.

Target	Conjugate	Host	Article-No.	Supplier	Dilution
KI67	-	Mouse	M724029-2	Agilent	1:100
SOX17	-	Rabbit	ab32034	Abcam	1:200
SOX2	-	Rat	14-9811-80	ThermoFisher	1:100
FoxA2/HNF3β	-	Rabbit	8186T	Cell Signaling	1:100
Mouse IgG	HRP	Rabbit	P0260	Dako	1:300
Rabbit IgG	HRP	Goat	P0448	Dako	1:300
RAT IgG	HRP	Donkey	A18739	ThermoFisher	1:300
Rabbit IgG	Alexa 488	Goat	ab150077	Abcam	1:300
Rat IgG	Cy3	Goat	A10522	ThermoFisher	1:200

Supplementary Data 1: TMM normalized min-max scaled expression levels were used as source data for generating figures 2 and 3.
