# Supplementary material for: Developmental progression continues during embryonic diapause in the roe deer
Source: Commun Biol. 2024 Mar 5;7:270. doi: 10.1038/s42003-024-05944-w (PMC10914810; doi:10.1038/s42003-024-05944-w)
Supplement: Supplementary file 2 — Description of Additional Supplementary Files [file 42003_2024_5944_MOESM2_ESM.pdf]

Description of Additional Supplementary Files

File name: Supplementary Data 1

Description: TMM normalized min-max scaled expression levels were used as source data for generating figures 2 and 3.
