# Supplementary material for: Developmental progression continues during embryonic diapause in the roe deer
Source: Commun Biol. 2024 Mar 5;7:270. doi: 10.1038/s42003-024-05944-w (PMC10914810; doi:10.1038/s42003-024-05944-w)
Supplement: Supplementary file 4 — Reporting Summary [file 42003_2024_5944_MOESM4_ESM.pdf]

nature portfolio

Corresponding author(s): Ulbrich, Susanne E

Last updated by author(s): 06.05.2023

Reporting Summary

Nature Portfolio wishes to improve the reproducibility of the work that we publish. This form provides structure for consistency and transparency in reporting. For further information on Nature Portfolio policies, see our Editorial Policies and the Editorial Policy Checklist.

Statistics

For all statistical analyses, confirm that the following items are present in the figure legend, table legend, main text, or Methods section.

n/a	Confirmed	
☐	☒	The exact sample size (*n*) for each experimental group/condition, given as a discrete number and unit of measurement
☐	☒	A statement on whether measurements were taken from distinct samples or whether the same sample was measured repeatedly
☐	☒	The statistical test(s) used AND whether they are one- or two-sided<br>*Only common tests should be described solely by name; describe more complex techniques in the Methods section.*
☐	☒	A description of all covariates tested
☐	☒	A description of any assumptions or corrections, such as tests of normality and adjustment for multiple comparisons
☐	☒	A full description of the statistical parameters including central tendency (e.g. means) or other basic estimates (e.g. regression coefficient) AND variation (e.g. standard deviation) or associated estimates of uncertainty (e.g. confidence intervals)
☐	☒	For null hypothesis testing, the test statistic (e.g. *F*, *t*, *r*) with confidence intervals, effect sizes, degrees of freedom and *P* value noted<br>*Give P values as exact values whenever suitable.*
☐	☒	For Bayesian analysis, information on the choice of priors and Markov chain Monte Carlo settings
☐	☒	For hierarchical and complex designs, identification of the appropriate level for tests and full reporting of outcomes
☐	☒	Estimates of effect sizes (e.g. Cohen's *d*, Pearson's *r*), indicating how they were calculated

Our web collection on statistics for biologists contains articles on many of the points above.

Software and code

Policy information about availability of computer code

Data collection	Olympus cellSens Standard, ZEN Black 2012 (light sheet), Illumina NovaSeq
Data analysis	R version 4.2.0 (packages and versions described in methods), Inkscape 1, ImageJ

For manuscripts utilizing custom algorithms or software that are central to the research but not yet described in published literature, software must be made available to editors and reviewers. We strongly encourage code deposition in a community repository (e.g. GitHub). See the Nature Portfolio guidelines for submitting code & software for further information.

Data

Policy information about availability of data

All manuscripts must include a data availability statement. This statement should provide the following information, where applicable:

- Accession codes, unique identifiers, or web links for publicly available datasets
- A description of any restrictions on data availability
- For clinical datasets or third party data, please ensure that the statement adheres to our policy

The raw transcriptome data analysed in this article has been previously published [31] and is accessible in NCBI's Gene Expression Omnibus (GEO) through GEO Series Accession No. GSE158806 (https://www.ncbi.nlm.nih.gov/geo/query/acc.cgi?acc=GSE158806). All custom code will be provided upon request.

Research involving human participants, their data, or biological material

Policy information about studies with human participants or human data. See also policy information about sex, gender (identity/presentation), and sexual orientation and race, ethnicity and racism.

Reporting on sex and gender	Humans, their data or biological material have not been used in this study.
Reporting on race, ethnicity, or other socially relevant groupings	Humans, their data or biological material have not been used in this study.
Population characteristics	Humans, their data or biological material have not been used in this study.
Recruitment	Humans, their data or biological material have not been used in this study.
Ethics oversight	Humans, their data or biological material have not been used in this study.

Note that full information on the approval of the study protocol must also be provided in the manuscript.

Field-specific reporting

Please select the one below that is the best fit for your research. If you are not sure, read the appropriate sections before making your selection.

☒ Life sciences ☐ Behavioural & social sciences ☐ Ecological, evolutionary & environmental sciences

For a reference copy of the document with all sections, see nature.com/documents/nr-reporting-summary-flat.pdf

Life sciences study design

All studies must disclose on these points even when the disclosure is negative.

Sample size	The samples were chosen to adequately represent the diapause and elongation period in the roe deer as far as this is possible given the field sampling strategy employed.
Data exclusions	In contrast to the publication (van der Weijden et al, 2021) from which the RNA-seq data was retrieved, all samples from in vivo collection were excluded due to the difference in the sampling procedure. In addition, data from one embryo was excluded due to unreasonably low DNA content.
Replication	Due to the samples originating from wild animals, exact biological replicates cannot be obtained.
Randomization	Any grouping was either performed based on morphological distinction or on hierarchical clustering. In the case of multiple batches of staining, samples were allocated such that each batch contained comparable number of samples and a variability representative of the one of the full sample set
Blinding	Due to the size of the embryo and the date of sampling, blinding was not possible during data collection. Each individual embryo was given an ID that did not allow inferring the origin, thus, data analysis was blinded regarding group allocation .

Reporting for specific materials, systems and methods

We require information from authors about some types of materials, experimental systems and methods used in many studies. Here, indicate whether each material, system or method listed is relevant to your study. If you are not sure if a list item applies to your research, read the appropriate section before selecting a response.

Materials & experimental systems

n/a	Involved in the study	
☐	☒	Antibodies
☒	☐	Eukaryotic cell lines
☒	☐	Palaeontology and archaeology
☐	☒	Animals and other organisms
☒	☐	Clinical data
☒	☐	Dual use research of concern
☒	☐	Plants

Methods

n/a	Involved in the study	
☒	☐	ChIP-seq
☒	☐	Flow cytometry
☒	☐	MRI-based neuroimaging

Antibodies

Antibodies used	See table 2:<br>Target - Conjugate Host Article-No. Supplier Dilution<br>KI67 - Mouse M724029-2 Agilent 1:100<br>SOX17 - Rabbit ab32034 Abcam 1:200<br>SOX2 - Rat 14-9811-80 ThermoFisher 1:100<br>FoxA2/HNF3β - Rabbit 8186T Cell Signaling 1:100<br>Mouse IgG HRP Rabbit P0260 Dako 1:300<br>Rabbit IgG HRP Goat P0448 Dako 1:300<br>RAT IgG HRP Donkey A18739 ThermoFisher 1:300<br>Rabbit IgG Alexa 488 Goat ab150077 Abcam 1:300<br>Rat IgG Cy3 Goat A10522 ThermoFisher 1:200
Validation	For none of the antibodies knock-out validation in roe deer was performed due to absence of adequate animal models or cellular expression systems;<br>KI67 antibody has been tested previously (see Rüegg et al., 2020);<br>SOX17, SOX2 and FOXA2 antibodies were tested for signal localization using sections of fetal and adult roe deer tissues;

Animals and other research organisms

Policy information about studies involving animals; ARRIVE guidelines recommended for reporting animal research, and Sex and Gender in Research

Laboratory animals	Laboratory animals have not been used in this study.
Wild animals	No animals were caught or killed specifically for these study. All samples were collected from roe deer shot by trained hunters in the course of regular huntings in Switzerland and southern Germany.
Reporting on sex	All embryos were retrieved from female animals. The sex of these embryos was not determined. The sex ratio of the embryos under analysis is presumed to be around 50%.
Field-collected samples	All samples were collected between September and January. After retrieval, the uteri were kept on ice and processed at ambient temperature until either snap frozen of fixed using formalin.
Ethics oversight	An ethical approval was not required, since all samples were collected at regular huntings from carcass remnants irrespective of the research question.

Note that full information on the approval of the study protocol must also be provided in the manuscript.
